# Acute liver injury following acetaminophen administration does not activate atrophic pathways in the mouse diaphragm

**DOI:** 10.1038/s41598-021-85859-2

**Published:** 2021-03-18

**Authors:** C. S. Bruells, P. Duschner, G. Marx, G. Gayan-Ramirez, N. Frank, T. Breuer, O. Krenkel, F. Tacke, J. C. Mossanen

**Affiliations:** 1 Department of Intensive and Intermediate Care, University Hospital Aachen, Aachen, Germany; 2 Department of Medicine III, University Hospital Aachen, Aachen, Germany; 3 Department of Anesthesiology, University Hospital Aachen, Aachen, Germany; 4 Laboratory of Pneumology, Katholieke Universiteit Leuven, Leuven, Belgium; 5 Department of Hepatology & Gastroenterology, Charité University Medical Center, Berlin, Germany

**Keywords:** Respiration, Molecular biology, Hepatology

## Abstract

N-acetyl-para-amino phenol (APAP, usually named paracetamol), which is commonly used for its analgesic and antipyretic properties may lead to hepatotoxicity and acute liver damage in case of overdoses. Released cytokines and oxidative stress following acute liver damage may affect other organs’ function notably the diaphragm, which is particularly sensitive to oxidative stress and circulating cytokines. We addressed this issue in a mouse model of acute liver injury induced by administration of APAP. C57BL/6J mice (each n = 8) were treated with N-acetyl-para-amino phenol (APAP) to induce acute drug caused liver injury and sacrificed 12 or 24 h afterwards. An untreated group served as controls. Key markers of inflammation, proteolysis, autophagy and oxidative stress were measured in diaphragm samples. In APAP treated animals, liver damage was proven by the enhanced serum levels of alanine aminotransferase and aspartate aminotransferase. In the diaphragm, besides a significant increase in IL 6 and lipid peroxidation, no changes were observed in key markers of the proteolytic, and autophagy signaling pathways, other inflammatory markers and fiber dimensions. The first 24 h of acute liver damage did not impair diaphragm atrophic pathways although it slightly enhanced IL-6 and lipid peroxidation. Whether longer exposure might affect the diaphragm needs to be addressed in future experiments.

## Introduction

Drug-induced liver injury represents a serious problem growing in prevalence and importance. Acute liver damage is induced by several toxic agents, of which N-acetyl-para-amino phenol (APAP, usually named paracetamol) is the most common drug responsible for toxic liver injury^1^. APAP is highly used due to its analgesic and antipyretic properties and while safe and effective at therapeutic doses, its overdose may lead to hepatotoxicity and acute liver damage. At therapeutic doses, most of APAP is metabolized by phase II conjugating enzymes and excreted as a non-toxic compound. The remaining APAP is degraded via the cytochrome p-450 system into a more toxic substance, N-acetyl-p-benzoquinone imine, whose toxic action can be prevented by glutathione^1,2^. In APAP overdose, glutathione scavenger reserves fade and, once depleted, the toxic metabolite damages liver cells, especially mitochondria where it leads to mitochondrial membrane permeabilization and dysfunction^3^.

Hence, acute liver injury can end as a systemic disease with decreasing liver synthesis and detoxification function and increasing cytokine production. Several authors pointed to the fact that acute liver injury is probably not an entity that remains limited to the liver^4,5^. In acute damage, the liver releases cytokines into the circulation^4,5^ and secondarily may influence other organs’ function and biology.

The diaphragm is particularly sensitive to oxidative stress and circulating cytokines^6,7^ and diaphragm weakness with muscle atrophy through activation of several proteolytic pathways and autophagy has been reported in situations associated with enhanced circulating cytokines and/or oxidative stress^8,9^.

We therefore hypothesized that acute liver injury may affect the diaphragm and induce atrophic signaling via protease-based proteolysis and autophagy. This issue was addressed in a mouse model of acute liver injury induced by administration of APAP.

## Methods

The study was approved by the appropriate governmental institution (Landesamt für Natur-, Umwelt- und Verbraucherschutz, LANUV, Germany, reference number: AZ 84-02.04.2014.A165) which granted the ethical approval for this study. The study was conducted in accordance with the principles for care and use of animals based on the Helsinki Declaration and the ARRIVE criteria.

### Induction of liver injury and animal model

C57BL/6J mice were housed under specific pathogen free conditions (Janvier labs, Le Genedt-Saint-Isle, France) and were divided into 3 groups (each n = 8): (1) acute liver injury sacrified at 12 h (APAP12), (2) acute liver injury sacrified at 24 h (APAP24), (3) an untreated control group (CTRL). Acute liver injury was induced as described in detail before^10,11^. Briefly, after a fasting period of 12 h, the animals received 250 mg/kg of APAP (Actavis Deutschland GmBh& CO. Kg, Langenfeld, Germany) by a single i.v. injection into a tail vein. After sacrifice, diaphragm was removed quickly and further processed (see below). Alanine aminotransferase (ALT) and aspartate aminotransferase activity (UV test at 37 °C) was measured in serum (Roche modular preanalytics system; Roche Diagnostics International AG, Rotkreuz, Switzerland).

### Diaphragm histology

Diaphragm strips were longitudinally embedded in formalin and cut in 10 µm thin slides. Staining was performed using hematoxylin and eosin as routine laboratory staining. Around 50 fibers of 3 animals of each group were used for assessment of cross-sectional areas using Image J software (Image J, NIH, USA).

### Protein extraction for electrophoresis and western blotting

Diaphragm tissue samples were homogenized 1:10 (weight volume^−1^) in 5 mmol Tris·HCl and 5 mmol EDTA buffer (pH at 7.5) containing a protease inhibitor cocktail (Roche, Basel, Swiss) and centrifuged at 1500×*g* for 10 min at 4 °C. After collection of the resulting supernatant, diaphragmatic protein content was assessed by the method of Bradford^12^, (Bio-Rad Laboratories Inc., Hercules, CA, USA). Proteins were detected with Chemiluminescent Peroxidase Substrate (Sigma-Aldrich, Overijse, Belgium), imaged with the Proxima 2850T imaging system (Isogen Life Technologies, De Meern, Netherlands) and analyzed using the TotalLab 1D software (Isogen Life Technologies, De Meern, Netherlands).

### Autophagy and Pi3K-Akt pathway

Autophagosome formation was assessed by measuring the conversion of LC3B-I to LC3B-II via immunoblotting. Proteins were separated on a 12% polyacrylamide gel and transferred onto a polyvinylidene fluoride (PVDF) membrane. Blots were incubated overnight at 4 °C with the primary antibody (# LC3A/B 4108S, Cell Signaling/Bioké, Leiden, The Netherlands) and subsequently with the appropriate secondary antibody (# P0217, Agilent, Heverlee, Belgium) for 1 h at room temperature. Data were expressed as LC3B-II/LC3B-I ratio.

Upstream signaling via Phosphatidyl-inositol-Kinase-3 (Pi3K) and Protein kinase B (AKT) was also measured by detecting the phosphorylated and non-phosphorylated form of Pi3K (Pi3K; #4249, Cell Signaling, Danvers USA) and of Protein kinase B (AKT; #4691, Cell Signaling, Danvers USA). Data were expressed as ratio between phosphorylated to total (non-phosphorylated) protein.

### Calpain-1 and caspase-3 activities

In vivo calpain-1 and caspase-3 activities were indirectly assessed, by measuring cleavage of αII-spectrin, a specific substrate for both calpain-1 and caspase-3. After separation of the proteins on a 10% polyacrylamide gel and transfer to PVDF membrane, the blot was incubated with the primary antibody against αII-spectrin (α-Fodrin FG6090, Enzo Life Science, Brussels, Belgium) and the suitable secondary antibody (P0260, Agilent, Heverlee, Belgium). Activities of calpain and caspase-3 were measured as the ratio between the densitometric values of their breakdown products to intact αII-spectrin^13^. The cleavage product of intact αII-spectrin by calpain gives a band at 150 kDa and a band at 120 kDa when cleaved by caspase-3 while intact αII-spectrin is detected at 260 kDa^14^. Active Caspase 3 was measured with their cleaved forms (Caspase-3: #C9662, Cell Signaling, Danvers, USA).

### Atrogin and MuRF-1

The activation of E3 ligases Atrogin and MURF-1 as markers of the ubiquitin–proteasome pathway was investigated by Western blotting using the antibodies: (Atrogin: ECM Biosciences, #AP2041, Versailles, USA; MURF-1: ABCAM, #AB77577, Eugene; USA).

### Oxidative stress: assessment of lipid peroxidation

Diaphragmatic 4-hydroxynonenal (4-HNE) was used as a marker of lipid peroxidation. After electrophoresis (7.5% polyacrylamide gel and blotting) polyclonal anti-4-HNE antibody (# MAB3249, R&D Systems, Abingdon, UK) was used as primary antibody and a polyclonal rabbit anti-mouse (# P0260, Agilent, Heverlee, Belgium) as secondary antibody. Data were expressed as a ratio between densitometric values of 4-HNE and Vinculin^15^.

### Inflammatory response: NF-ĸB subunits

The p50 and p 65 subunits of NFkB were assessed by Western blotting using P50/P65: #4764 and phospho-P65: #3031, Cell-Signaling, Danvers, MA, USA.

### RNA preparation and quantitative real-time PCR (qPCR)

RNA from murine diaphragm samples were extracted with TRIZOL. 2 µg purified RNA were reverse transcribed (Maxima H Minus Kit with dsDNase, Thermo Fisher #K1682). qPCR was run on a StepOne Plus instrument (LifeTechnologies) using Power SYBR Green Master Mix (Applied Biosystem #4368706). For messenger RNA (mRNA) analysis, inflammatory and proteolytic marker expression was normalized with ribosomal protein S7. RNA expression was analyzed by the ΔΔ C_T_ method. The following primers were used (see Table 1).

**Table 1:** Primers for mRNA detection.

TNF-a for	CCACGTTGTAGCCAATGTC
TNF-a rev	GAGTAGATGAGGTACAGCCC
IL 6 for	GGTTCAATCAGGAGACCTGC
IL 6 rev	GCTTTGTCTGGATTCTTTCCC
IL-10 for	GCATCCACTTCCCAACCAG
IL-10 rev	ACCCAGGTAACCCTTAAAGTC
Casp3 for	TCATAATTCAGGCCTGCCGA
Casp3 rev	GCTGCACAAAGTGACTGGA
Casp7 for	CAT GGA TTT CCA GAA GAT GGG
Casp7 rev	CTG TCA GAT CCT TTA TGG GTG
IL-1ß1 for	TGAAGAAGAGCCCATCATCCT
IL-1ß1 rev	TCATGCAGAACACCACTTCTC
S7 for	AAATAAGCAGAAGCGTCCCA
S7 rev	AATGTTTCAACCTTGTGCTCC

*TNF* tumor necrosis factor, *IL* interleukin, *Casp* Caspase, *S7* ribosomal protein 7.

### Statistical analysis

Population distribution was assessed with the Kolmogorov–Smirnov test. If this test showed normal distribution of data, comparisons between groups for each dependent variable were made by ANOVA, if normal distribution was not present, by a Kruskall Wallis test. If the group effect was significant, a Dunn’s multiple comparisons test was used for pairwise comparisons between all groups. Data are shown as means ± standard deviation (SD). All statistical tests are two-tailed, significance was established at p < 0.05 (GraphPad Prism 6.0, La Jolla, CA, USA).

## Results

### Liver injury characteristics

**Figure 1:**
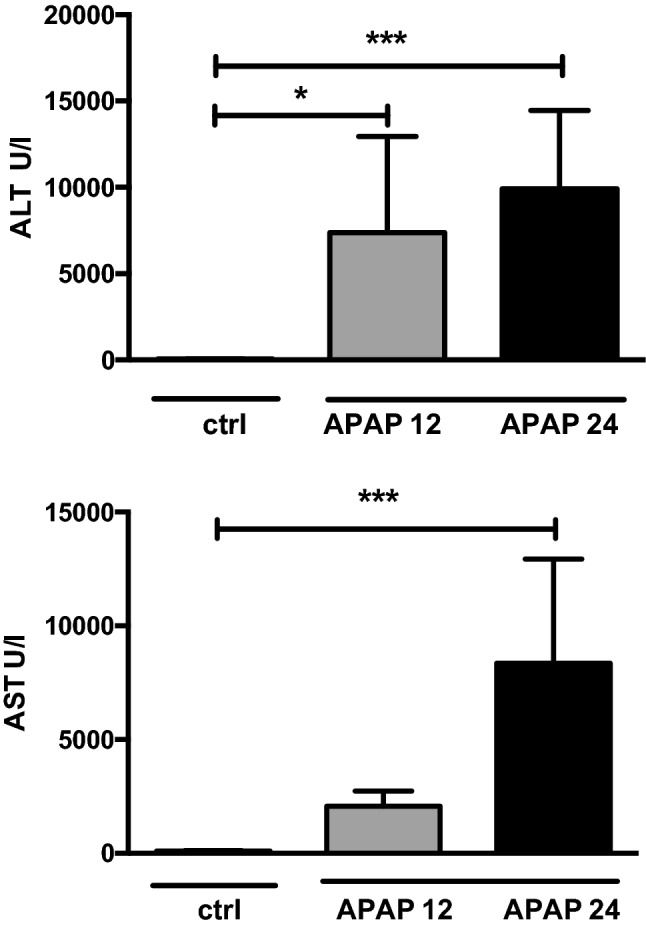
Serum levels of liver enzymes: alanine amino-transferase (ALT, upper panel) and aspartate aminotransferase activity (AST, lower panel) 12 h (APAP12) and 24 h (APAP24) after administration of N-acetyl-para-amino phenol (APAP) compared to controls (ctrl). The increase in liver enzymes indicated severe liver injury during the experiment. Values are mean and standard deviation expressed in U/l. * and *** indicate significance p < 0.001 or p < 0.0001 respectively.

Serum levels of ALT increased significantly and similarly 12 and 24 h after APAP administration compared to control (ctrl vs APAP 12 p = 0.01; ctrl vs APAP p < 0.001) while serum levels of AST increased significantly but only 24 h after APAP administration (ctrl vs APAP 24 p < 0.001) (see Fig. 1).

### Histologic assessment of diaphragm atrophy

No differences in muscle fiber size could be detected between the experimental groups (see histogram as Fig. S1 in the Supplemental File 1).

### Autophagy and Pi3K and AKT phosphorylation

**Figure 2:**
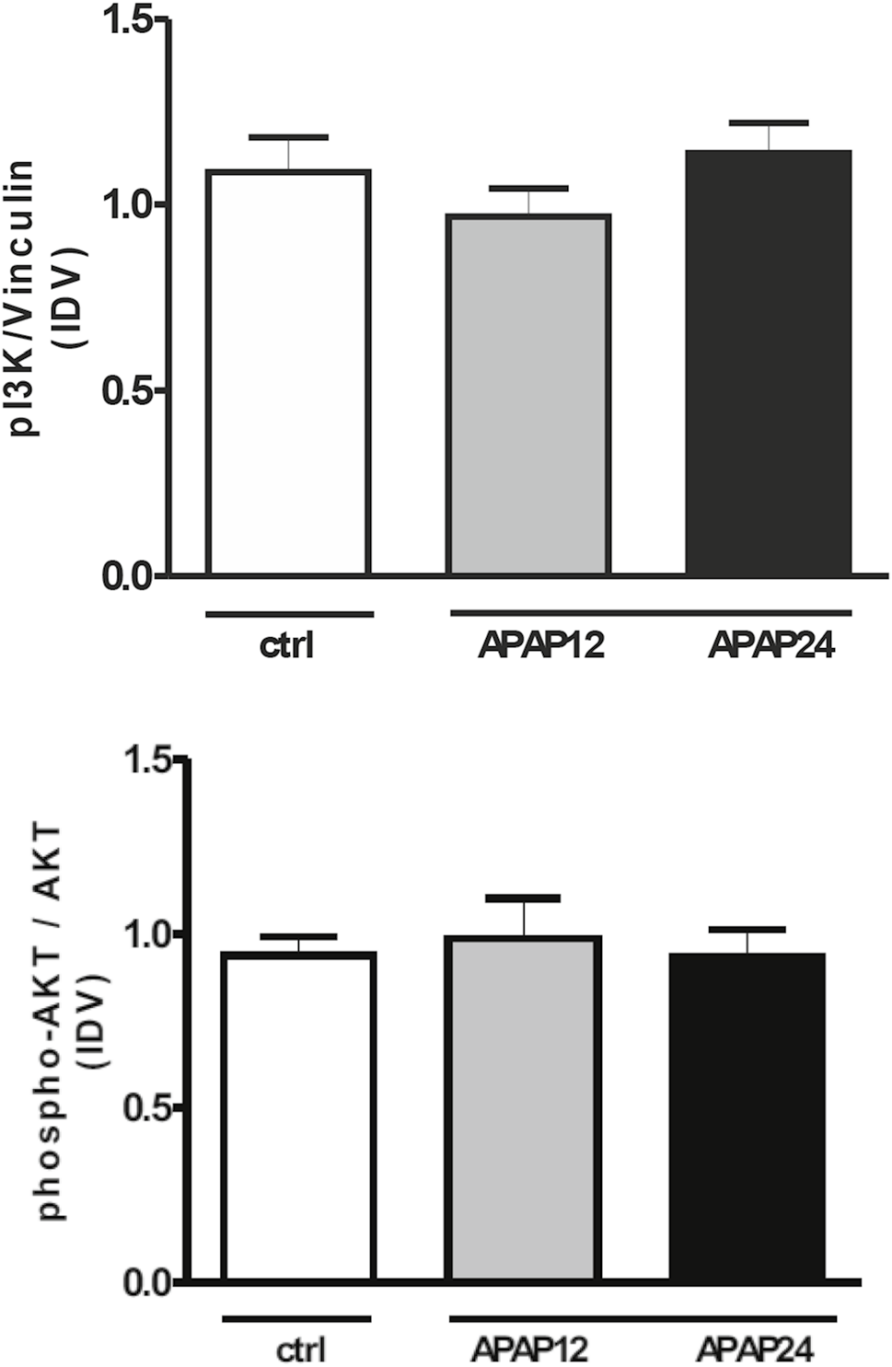
Changes in upstream signaling of protein synthesis. *Upper panel* Amount of Pi3K 12 or 24 h after APAP treatment. *Lower panel* changes of phosphorylated AKT vs AKT 12 or 24 h after APAP treatment. *Pi3K* phosphatidyl-inositol Kinase 3; AKT Protein kinase B; there is no change in these parameters. *APAP 12* N-acetyl-para-amino phenol 12 h treatment; *APAP 24* N-acetyl-para-amino phenol 24 h treatment, *ctrl* controls, *IDV* integrated density value. Values are mean and standard deviation.

**Figure 3:**
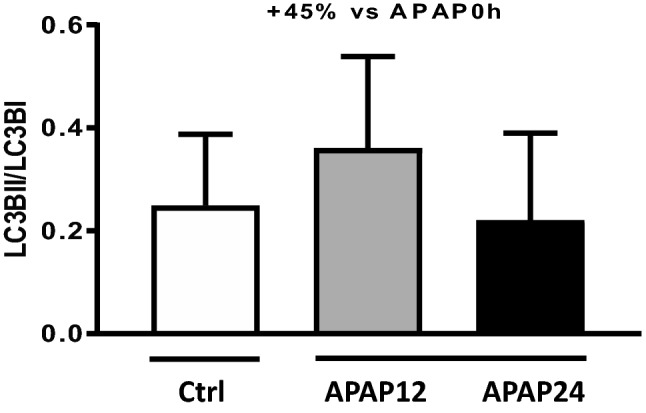
LC3B-I to LC3B-II ratio in control animals and after treatment. There is an increase in autophagosome formation after 12 h, but this increase fails to reach statistical significance. *APAP 12* N-acetyl-para-amino phenol 12 h treatment, *APAP 24* N-acetyl-para-amino phenol 24 h treatment, *ctrl* controls; Values are mean and standard deviation.

The amount of Pi3K was not significantly changed 12 or 24 h after treatment (see Fig. 2 upper panel). Downstream, p-AKT/AKT was also not alter by intervention (see Fig. 2 lower panel). There were no changes in downstream conversion of LC3B-I to LC3B-II (see Fig. 3). See corresponding blots in Supplemental File 1, Figs. S6 and S10 as well as in Supplemental File 2, Figs. S1 and S6.

### Proteolytic activation

Expression of Caspase 3 mRNA and Caspase 7 mRNA was not altered after 12 or 24 h compared to untreated controls. The protein levels of Pro-Caspase 3/Caspase 3 did not change at any time points compared to controls (mRNA data see Supplemental Fig. S3).

Caspase 3 and calpain-1 protein (measured as the amount of the specific breakdown products of α-II spectrin) were significantly decreased after 24 h after APAP intoxication vs ctrl (caspase 3: − 45%, p = 0.005; calpain 1: − 45%, p = 0.02) (see Fig. S4 in Supplement 1, as well as the corresponding blots in Supplemental File 1, Fig. S7, Supplemental File 2, Fig. S2).

### Activation of the Ubiquitin proteasome system

Downstream levels of Ubiquitin E3 ligases Atrogin and MURF did not differ between ctrl and the APAP treated animals (Fig. S5 in Supplement, as well as the corresponding blots in Supplemental File 1, Fig. S7, Supplemental File 2, Fig. S3).

### Diaphragm inflammatory response

**Figure 4:**
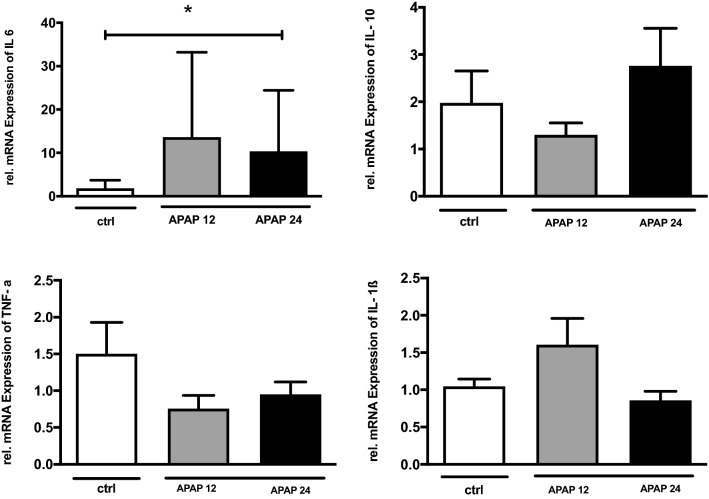
Inflammatory response in diaphragm tissue. IL-6, IL-10, TNFalpha and IL-1b mRNA expression relative to ctrl demonstrating a significant increase in IL-6 after *APAP 12* N-acetyl-para-amino phenol 12 h treatment; *APAP 24* N-acetyl-para-amino phenol 24 h treatment compared to control (ctrl). Values are mean and standard deviation *p < 0.02.

Levels of the NfκB subunits p50 and p65 were not altered in the different groups (see Fig. S6 in Supplement, as well as the corresponding blots in Supplemental File 1, Fig. S8 and Supplemental File 2, Fig. S4). The mRNA expression of IL-10, TNF-α, IL1-β did not change significantly while mRNA levels of IL-6 were significantly elevated 24 h after APAP exposure vs ctrl (p = 0.02) (see Fig. 4).

### Oxidative stress

**Figure 5:**
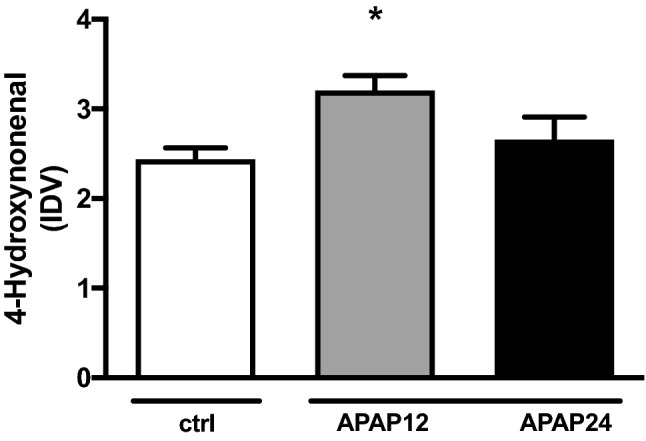
Lipid peroxidation in diaphragm tissue in interventional groups and control animals. 12 h after APAP exposure, there is a significant increase in lipid peroxidation. *4-HNE* 4-Hydroxdy-non-enal, *APAP 12* N-acetyl-para-amino phenol 12 h treatment, *APAP 24* N-acetyl-para-amino phenol 24 h treatment, *ctrl* controls; Data are normalized by vinculin and expressed as mean and SD. *IDV* integrated density value; *indicates significance p < 0.05.

Enhanced lipid peroxidation was present in the diaphragm 12 h after APAP exposure compared to controls (+ 30%, p = 0.02), while this increase failed to reach statistical significance after 24 h (see Fig. 5). The blots are displayed in Supplemental File 1, Fig. S9 and Supplemental File 2, Fig. S5.

## Discussion

This study showed that in mice, during the acute phase of liver injury, no activation of the proteolytic or autophagic pathways and no atrophy occurred in the diaphragm while lipid peroxidation was elevated and IL-6 was the only inflammatory marker to be upregulated inside the diaphragm. The effects on the diaphragm are discussed in detail below.

The APAP model to induce acute liver failure is a well-established model. In previous studies, we have proven that induction of necrosis in hepatocytes after administration of APAP leads to consistent results within groups. Analysis of immune cells and their modulation showed that the model induces “two hits” during acute liver injury generated by APAP^10^. Hepatocyte necroses are induced directly by the toxic metabolite NAPQI. Otherwise, severity of the liver injury and dimension of necrosis are affected by a massive immune cell infiltration into the liver caused by the initial damage^3^. Until know it is still ambiguous which part of the immune response and which immune cells respectively aggravate or attenuate liver injury at the different stages of the disease^16^.

We detected significant changes of liver enzymes’ serum levels and necrotic areas inside liver tissue, pointing to a profound damage of hepatocytes. Central clinical signs of the acute liver failure are prolonged prothrombin time/INR, declined mental function, vasodilation and a systemic inflammatory response syndrome due to the high immunological impact of the liver^4^. Summarily, acute liver failure results in multi-organ failure (Acute liver failure)^17^, although depending on etiology and duration course of illness is variable. In APAP and ischemia induced ALF the acute phase will be measured in hours and we expect systemic effects during the experimental time chosen in our model.

Inflammatory response to acute liver injury has been described in humans and several cytokines in drug induced liver injury were elevated in serum^18^. Blazka and colleagues revealed an immediate response of TNFα and IL1β after APAP induced liver injury induction with systemic response^19^. IL-6 can even more act in several different ways in muscle and is produced during exercise^20^ or diaphragm overload^21^. Moreover, Janssen et al. demonstrated severe muscle atrophy in the diaphragm after IL-6 administration into the circulation. However, in diaphragm the influence of inflammation on its function has been under discussion: Blocking of toll-like receptor-4 in mice in a disuse model could decrease the amount of autophagic activation and IL-6 production and ameliorate the reduction of myosin heavy chain protein^22^. Possibly, the animals had an increase in the work of breathing due to metabolic disturbances after liver injury, which might explain this increase in IL-6 in a quasi-physiologic response to overload.

Summarily, we could not detect a profound secondary response inside the diaphragm in the following 12 h or 24 h after liver injury induction except for enhanced levels of IL-6 that were not associated with changes in NFκB subunits or other inflammatory cytokines.

Oxidative stress is a key factor in the pathogenesis of ICU-acquired diaphragmatic weakness^5^ with mitochondria and to a smaller extent oxidases functioning as major sources of ROS^23–25^. In diaphragm, the increase of ROS interacts with the activity of main proteolytic pathways caspase 3 and calpain-1 as well as the autophagic system, so that blocking ROS results in an amelioration of atrophy via autophagic and proteolytic pathways^23,24,26,27^. In our model, we could detect a significant transient increase in the formation of lipid peroxidation (4 HNE) after 12 h of APAP induced liver injury. This increase abandoned in the 24 h APAP group, which could indicate a quick removal of oxygenated structures, as we have detected in a former experiment^28^. In our actual study, we did not distinguish further which source (mitochondria and/or oxidases) was responsible for the increase in oxidation. Importantly, we did not found a link between this increase in lipid peroxiadation and atrophy inducing pathways, although in disuse or sepsis this has been proven^26,27^. Possibly the amount of oxidative stress did not reach the level to induce proteolytic Ca-dependent proteases or autophagy.

In several diseases, proteolysis is activated inside the diaphragm and modulated by different pathways: Calcium dependent protease-based proteolysis via caspase 3 and calpain 1 have been described during sepsis, disuse (VIDD) and in chronic lung disease^29–31^. Caspase 3 acts a key protease, in conjunction with calpain 1, to break up the sarcomeric structures and allow downstream proteasome to further degrade the proteins^32,33^. Neither mRNA levels nor protein expression of key calcium-dependent proteases were enhanced in the diaphragm despite the presence of clear liver injury. Even more, the downstream ligases MURF and MAFbx remained unaltered in the interventional groups, although these ligases are involved in the further degradation of muscle protein after break-up by caspases^31^ and are usually activated during muscle atrophy, inflammation or oxidative stress^30^. In summary, proteolytic activation in the diaphragm by acute liver injury does not seem to be present in our model.

Autophagy is an important proteolytic pathway targeting cellular components associated with muscular atrophy via autophagosomes, which form double layer structures of which LC3 is a core molecule^27,34^. Upstream, Protein kinase B (AKT) and PI3K regulate via the Forkhead-Box-O1 transcription factor the formation of autophagosome building proteins, like LC3B.

The diaphragm is susceptible to slightest disturbances in the ratio of protein synthesis and proteolysis, so that even 12 h of disuse affect this balance^35^. Transformation from LCB3BI into LC3B-II indicates the activation of autophagosomes^30^ and Hussain and colleagues described this pathway as one major diaphragmatic pathway in disuse^36^ which is also active in sepsis and linked to oxidative stress^30^. In addition, the autophagy pathway can modulate protein synthesis through the mammalian target of rapamycin (mTOR) and serves therefore as a switch between anabolic and catabolic states^37^.

During liver injury as in our model, autophagy assessed by the ratio of LC3BII/LC3BI and by the upstream molecules was not activated in the diaphragm.

This study has, however, some limitations that should be taken into account. According to the goal of the study, 12 and 24 h were specifically chosen as time point measures after liver injury in order to address the early phase effects of liver injury on the diaphragm. We, however, acknowledge that later time point measures may be relevant to address the secondary complications reported to arise days after acute liver injury in humans. Further studies are needed to address the influence of acetaminophen induced liver injury on diaphragm dysfunction at later time points and whether known effects on lung function (e.g. airway hyper-reactivity, asthma, etc.) may add to these changes^38^. Those were in this study beyond the scope.

## Conclusion

In contrast to our hypothesis, acute liver injury induced by APAP in mouse did not induce atrophy or activate proteolysis via calcium dependent proteases or via ubiquitin proteasome pathway, neither it did stimulate autophagy. However, it resulted in enhanced diaphragm lipid peroxidation and upregulation of IL-6 which, on long-term, might be detrimental for the diaphragm. Future research might focus on long term models of liver injury to see whether the diaphragm would be affected.

## Supplementary Information

Supplementary Information 1.

Supplementary Information 2.
