# Supplementary material for: Acute liver injury following acetaminophen administration does not activate atrophic pathways in the mouse diaphragm
Source: Sci Rep. 2021 Mar 18;11:6302. doi: 10.1038/s41598-021-85859-2 (PMC7973759; doi:10.1038/s41598-021-85859-2)
Supplement: Supplementary file 1 — Supplementary Information 1. [file 41598_2021_85859_MOESM1_ESM.docx]

# Acute liver injury following acetaminophen administration does not activate atrophic pathways in the mouse diaphragm

Bruells CS^1,3*^, Duschner P^1,3^, Marx G^1^, Gayan-Ramirez G^4^, Frank N^1^, Breuer T^1^, Krenkel O^2^, Tacke F^5^, Mossanen JC^1,2^.

1 Department of Intensive and Intermediate Care, Aachen University hospital Aachen, Aachen Germany

2 Department of Medicine III, University Hospital Aachen, Aachen, Germany

3 Department of Anesthesiology, Aachen university hospital, Aachen Germany

4 Laboratory of Pneumology, Katholieke Universiteit Leuven, Leuven, Belgium

5 Department of Hepatology & Gastroenterology, Charité University Medical Center, Berlin, Germany

# Supplemental file 1:

Figure S1: Diaphragm hematoxylin and Eosin staining (upper panels A-C) and histogram of the diaphragm fiber cross-sectional area (CSA) (lower panel) of the different groups. Panel A and blue bars: ctrl, Panel B and orange bars: APAP12, Panel C and grey bars: APAP24.Fiber CSA are expressed in square micrometer; picture taken by the author

Figure S2: relative mRNA expression compared to S7 of Caspase-3 and Caspase-7 mRNA. Values are mean and standard deviation

Figure S3: Changes of specific breakdown product αII spectrin as markers of Caspase-3 or Calapin-1 activity.

APAP 12: N-Acetyl-Para-Amino Phenol 12 hours treatment; APAP 24: N-Acetyl-Para-Amino Phenol 24 hours treatment; ctrl: controls; IDV: integrated density value. Values are mean and standard deviation; blots see figure S7

Figure S4: Levels of MURF-1 and Atrogin-1 in the different experimental groups.

APAP 12: N-Acetyl-Para-Amino Phenol 12 hours treatment; APAP 24: N-Acetyl-Para-Amino Phenol 24 hours treatment; ctrl: controls; IDV: integrated density value. Values are mean and standard deviation; blots see figure S7

Figure S5: Changes of NfκB subunits p50 and p65 at the investigated timepoints. APAP 12: N-Acetyl-Para-Amino Phenol 12 hours treatment; APAP 24: N-Acetyl-Para-Amino Phenol 24 hours treatment; ctrl: controls; IDV: integrated density value. Values are mean and standard deviation; blots see figure S8.

Figure S6: Blot membranes at timepoint ctrl, APAP12 and APAP24 for AKT, pAKt and PI3K and Vinculin as loading control. Top: ctrl; Middle panel: APAP 12; bottom panel APAP 24; Membranes were cut before specific antibody was added to save samples and antibody; See also Supplemental file 2 without markers

Figure S7: Blot membranes at timepoint ctrl., APAP12, and APAP24 for a-II-spectrin, MURF, Atrogin and active Caspase 3 and Vinculin as loading control. Top: ctlr; Middle panel: APAP 12; bottom panel APAP 24; Membranes were cut before specific antibody was added to save samples and antibody

Figure S8: Blot membranes at timepoint ctrl., APAP12 and APAP24 for p50, Pp50, p65 and Pp65 . Top: ctrl.; Middle panel: APAP 12; bottom panel APAP 24; Membranes were cut before specific antibody was added to save samples and antibody


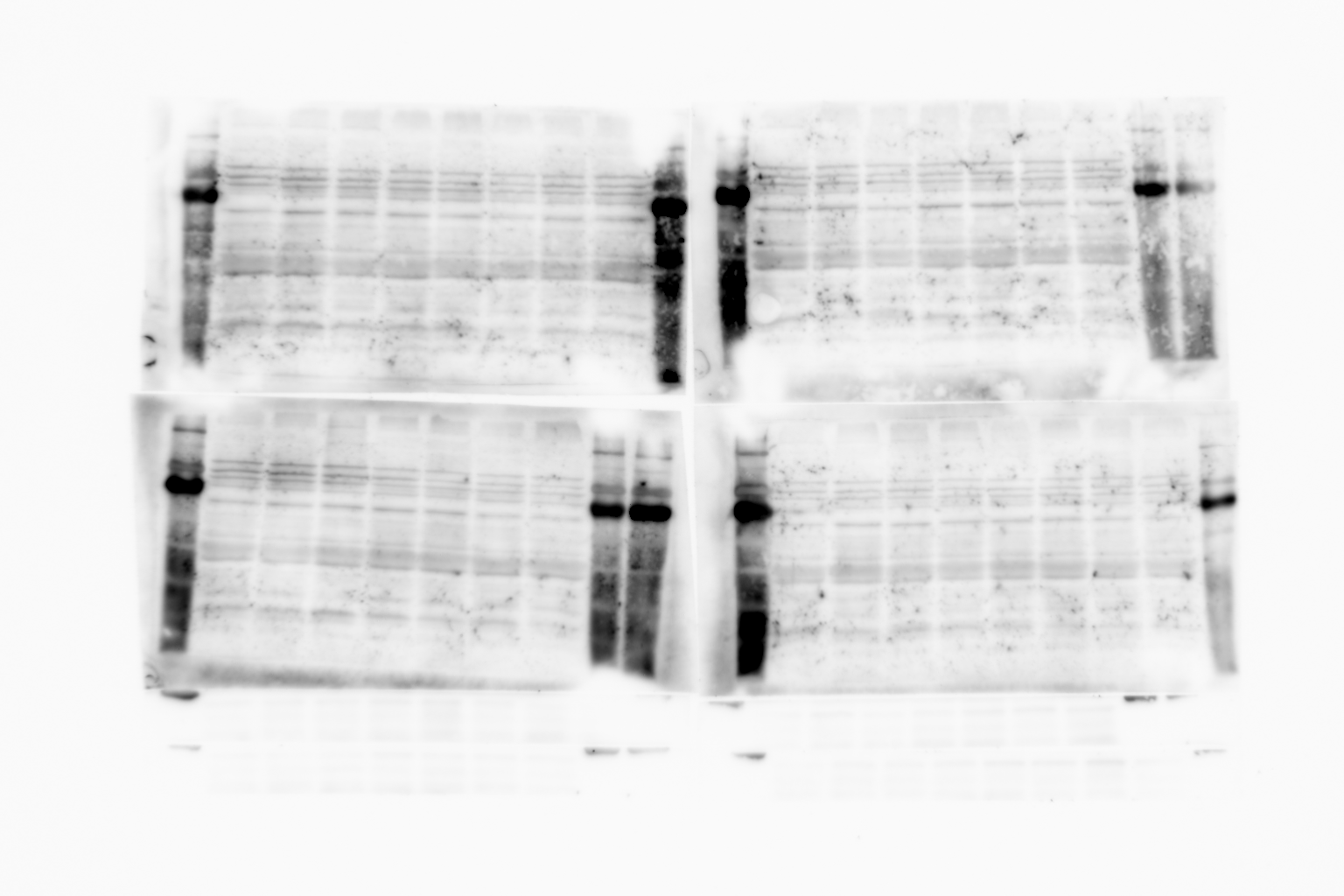


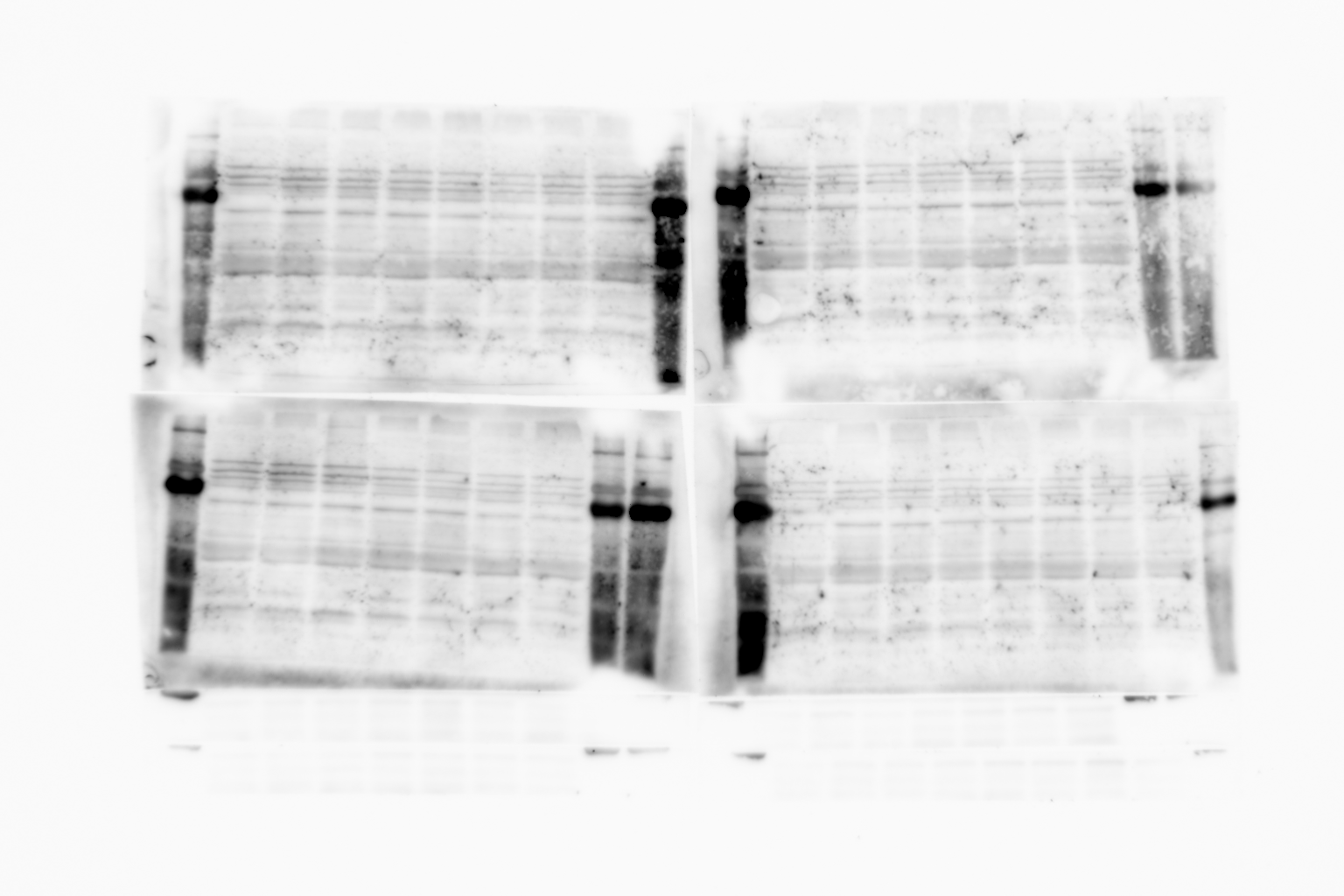


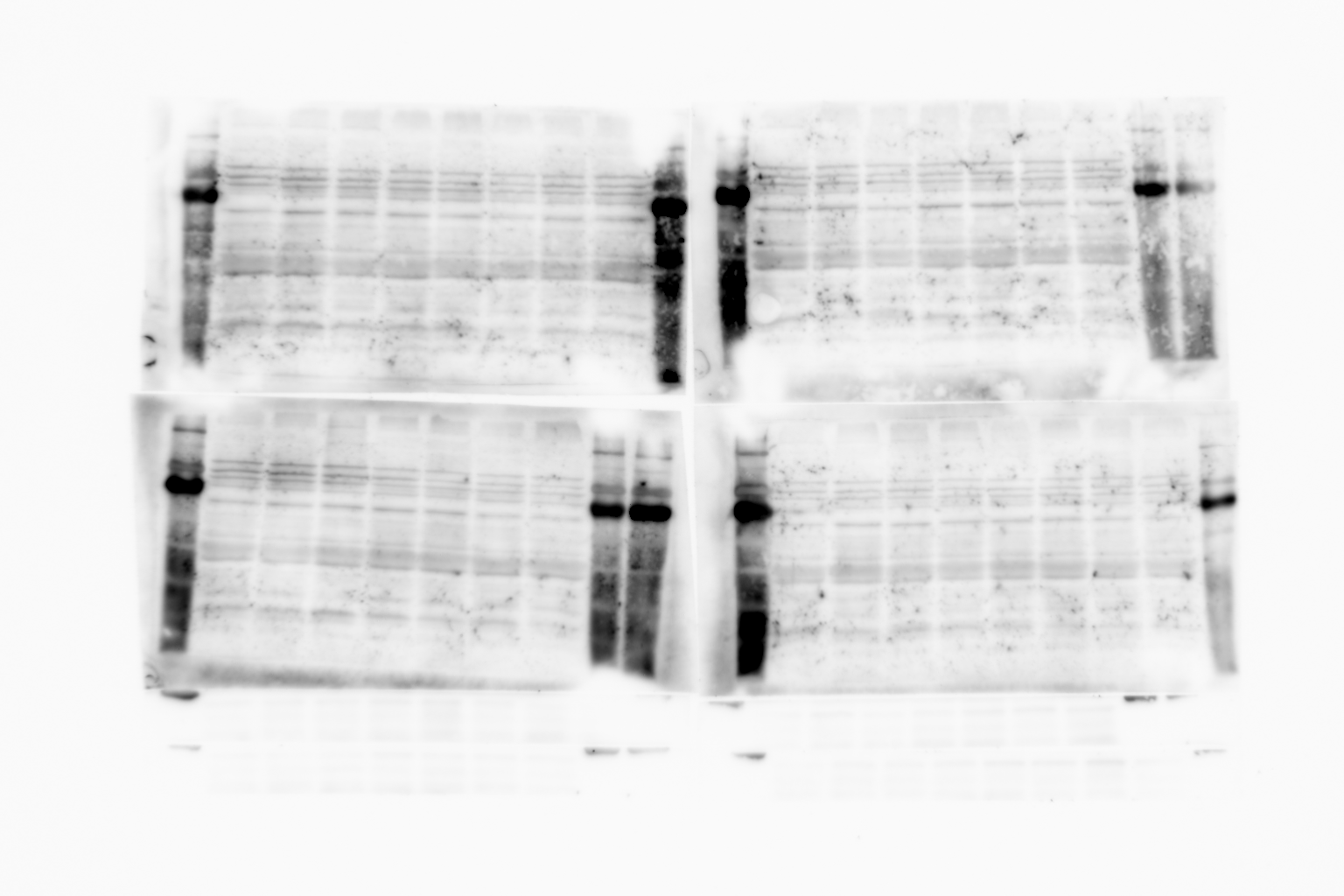


Figure S9: Blot membranes at timepoint ctrl., APAP12 and APAP24 for 4HNE. Top: ctrl.; Middle panel: APAP 12; bottom panel APAP 24; Membranes were cut before specific antibody was added to save samples and antibody

Figure S10: Blot membranes for the evaluation of LC3B; Please note, that these investigation was undertaken in the lab of pneumology in Leuven, so that the blot composition differs from the Aachen lab.
