# Supplementary material for: Acute liver injury following acetaminophen administration does not activate atrophic pathways in the mouse diaphragm
Source: Sci Rep. 2021 Mar 18;11:6302. doi: 10.1038/s41598-021-85859-2 (PMC7973759; doi:10.1038/s41598-021-85859-2)
Supplement: Supplementary file 2 — Supplementary Information 2. [file 41598_2021_85859_MOESM2_ESM.docx]

## Acute liver injury following acetaminophen administration does not activate atrophic pathways in the mouse diaphragm

Bruells CS^1,3*^, Duschner P^1,3^, Marx G^1^, Gayan-Ramirez G^4^, Frank N^1^, Breuer T^1^, Krenkel O^2^, Tacke F^5^, Mossanen JC^1,2^.

1 Department of Intensive and Intermediate Care, Aachen University hospital Aachen, Aachen Germany

2 Department of Medicine III, University Hospital Aachen, Aachen, Germany

3 Department of Anesthesiology, Aachen university hospital, Aachen Germany

4 Laboratory of Pneumology, Katholieke Universiteit Leuven, Leuven , Belgium

5 Department of Hepatology & Gastroenterology, Charité University Medical Center, Berlin, Germany

Supplemental file 2

The file contents the plain blot pictures of all blots undertaken in this study- without markers etc. Please note that membranes may be shorter sometimes: this is due to the fact that we saved antibody and sample by simply using smaller pieces of the whole membranes. Pictures of these very blots with a ladder description are found in supplementals file 1. In the beginning of the study we measured the time point at 6 hours, we later decided to remove this timepoint from the study. Nevertheless, all blots are displayed in the same order:

APAP 0 is in the upper left corner (which has been renamed in the paper as “ctrl.”, APAP 6 (removed from the study) in the upper right, APAP 12 in the lower left corner and APAP 24 in the lower right corner.


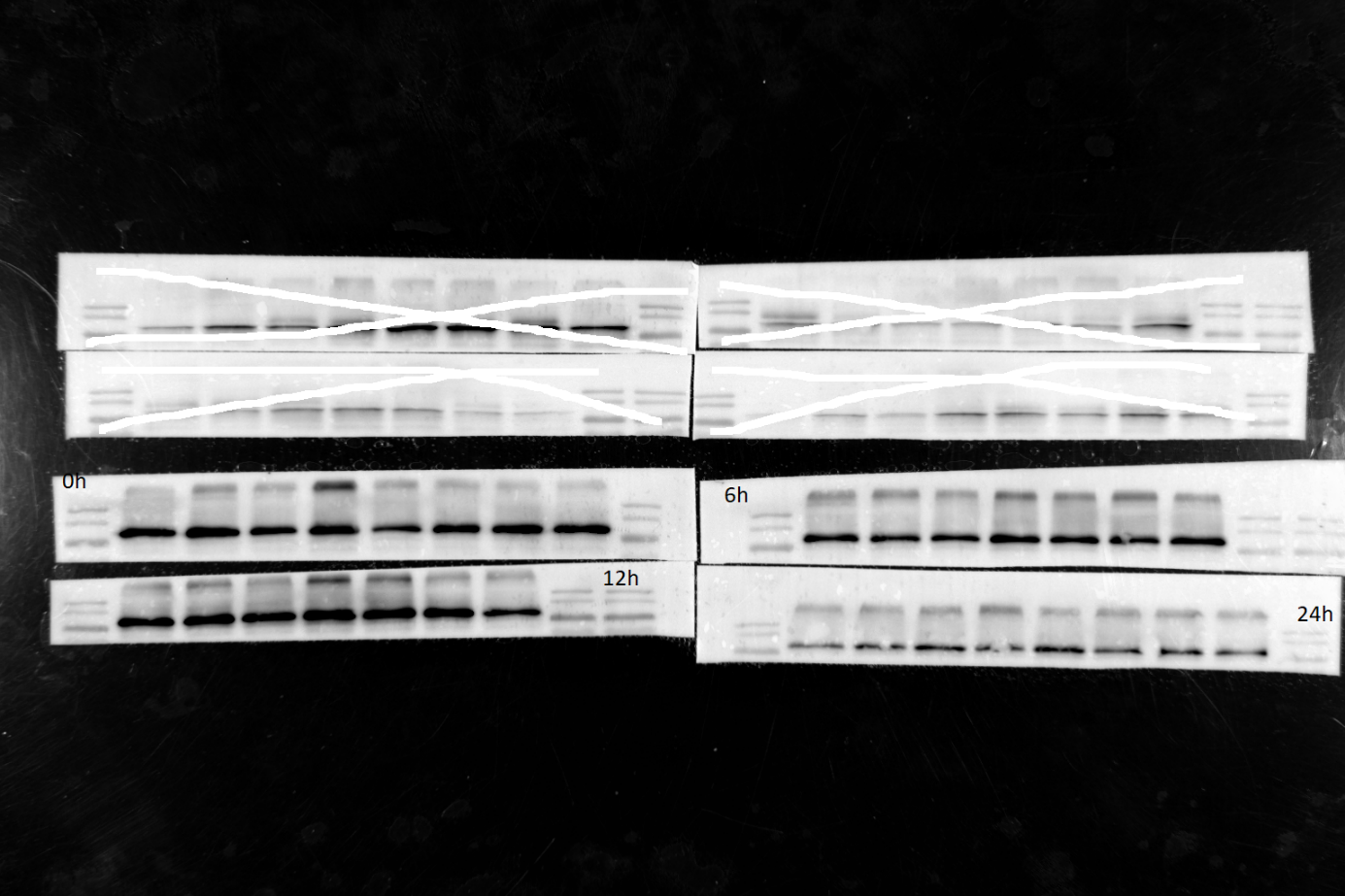


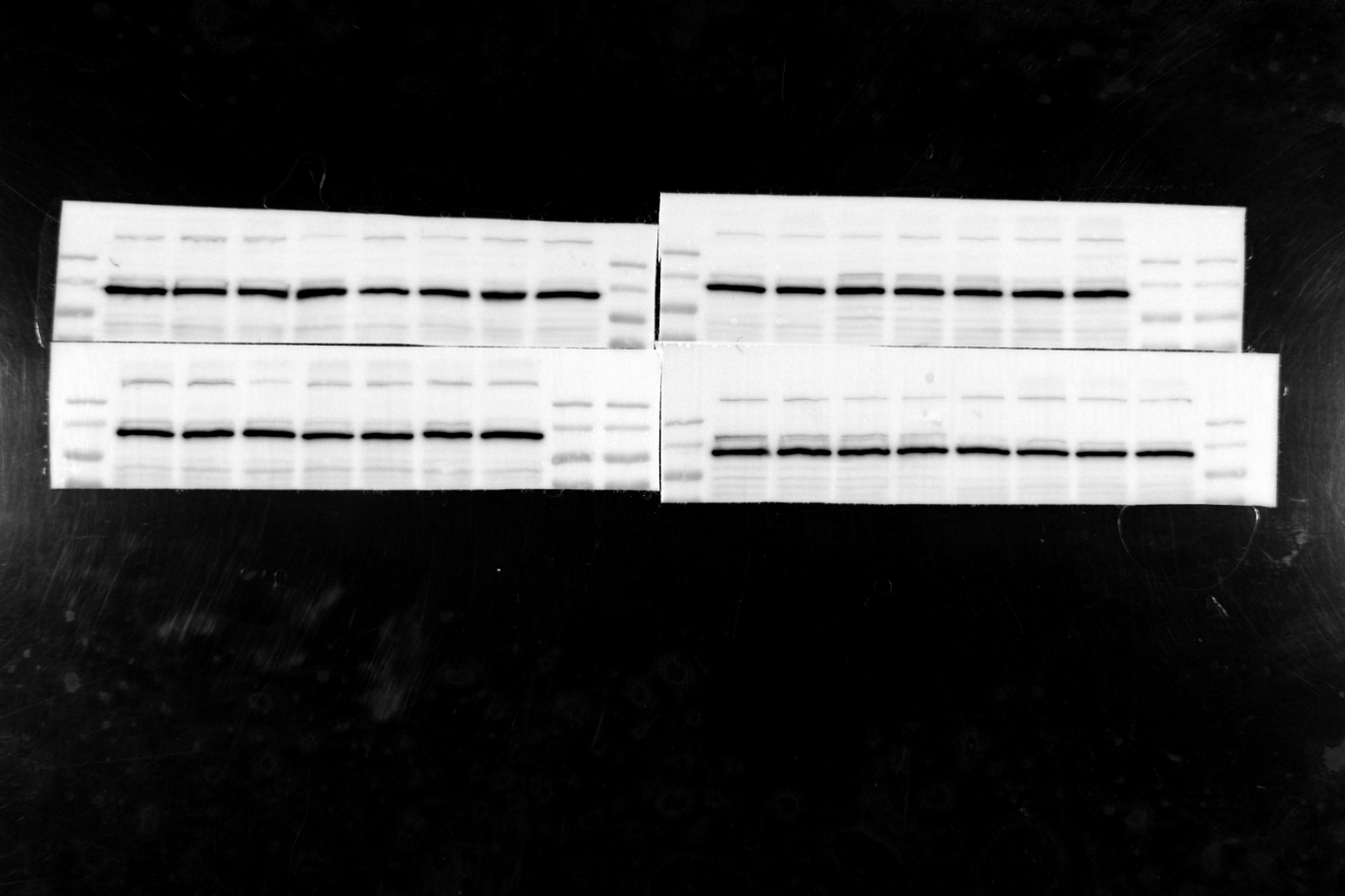


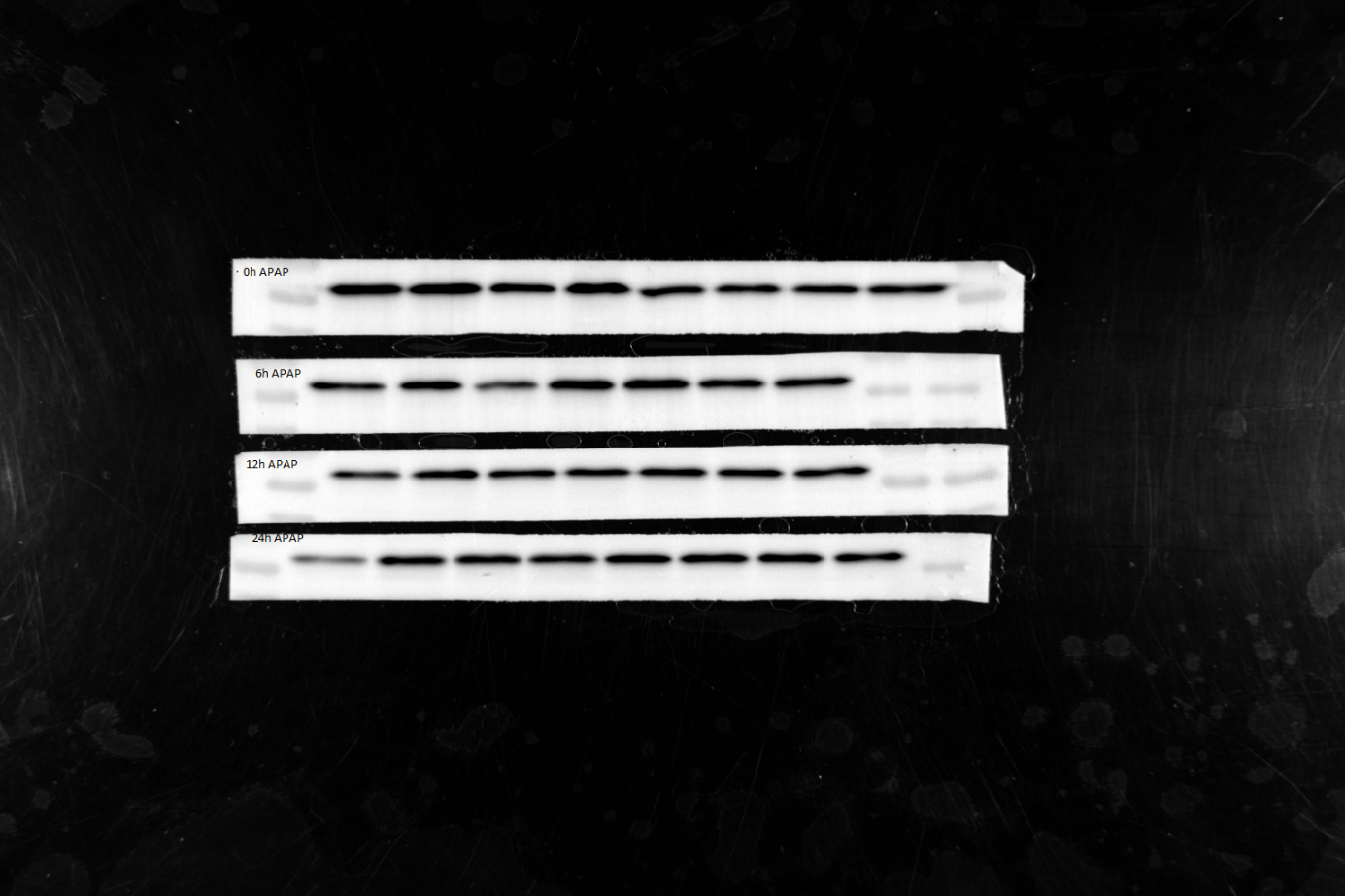


Figure S1: Blots of Pi3K (upper panel), AKT/pAKT (middle panel) and Vinculin (bottom).

Ctrl is in the upper left corner, APAP 6 (removed from the study) in the upper right, APAP 12 in the lower left corner and APAP 24 in the lower right corner.


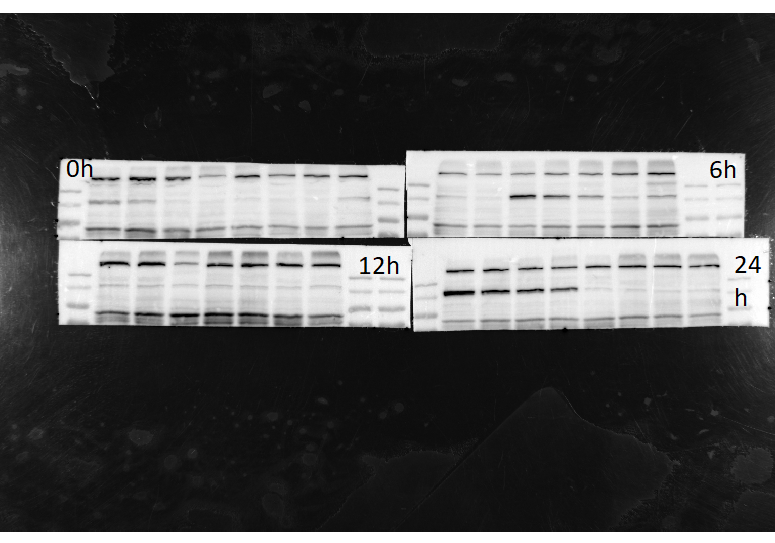


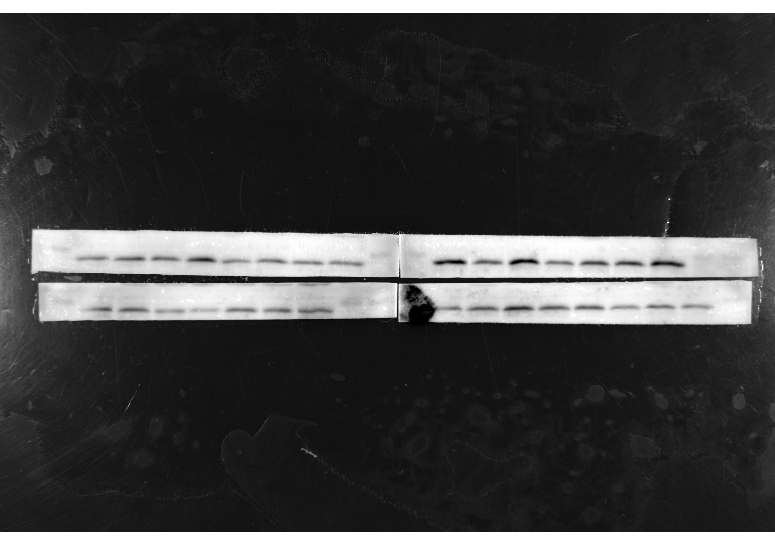

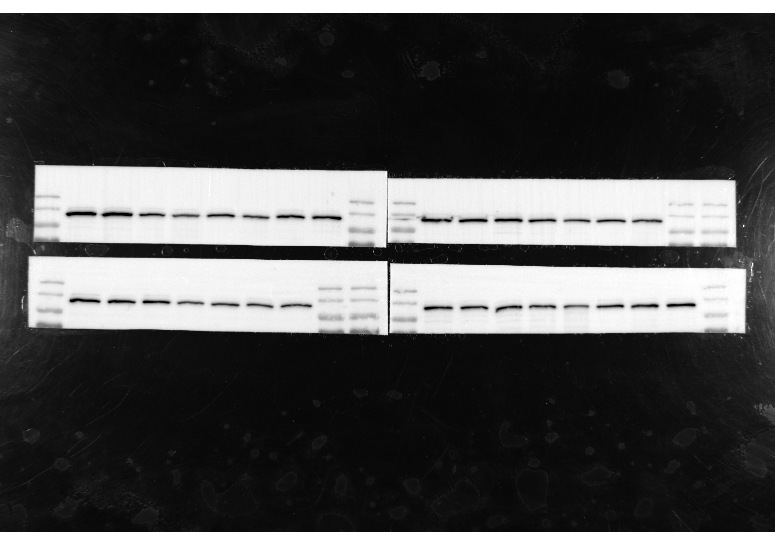


Figure S2: Plots of alpha II-spectrin (top) and Caspase 3 (middle) including the loading control (Vinculin, bottom). Please note that all blots are displayed in the same order as given in the top picture.

Ctrl. is in the upper left corner, APAP 6 (removed from the study) in the upper right, APAP 12 in the lower left corner and APAP 24 in the lower right corner.


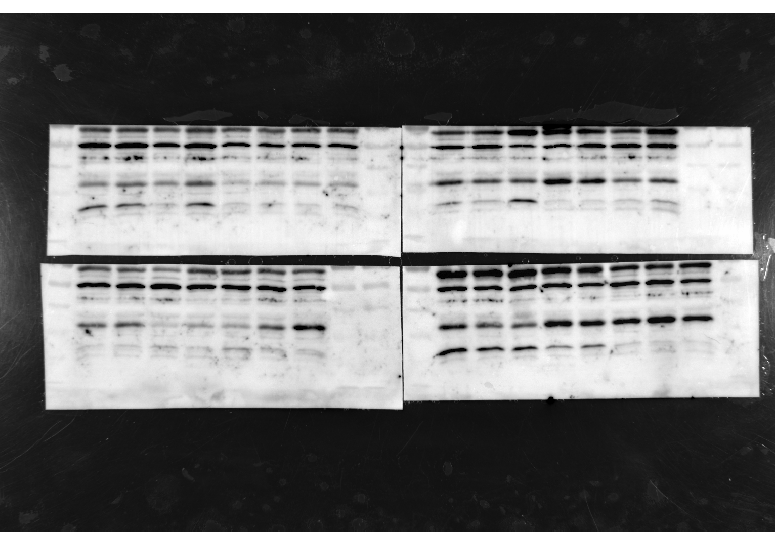


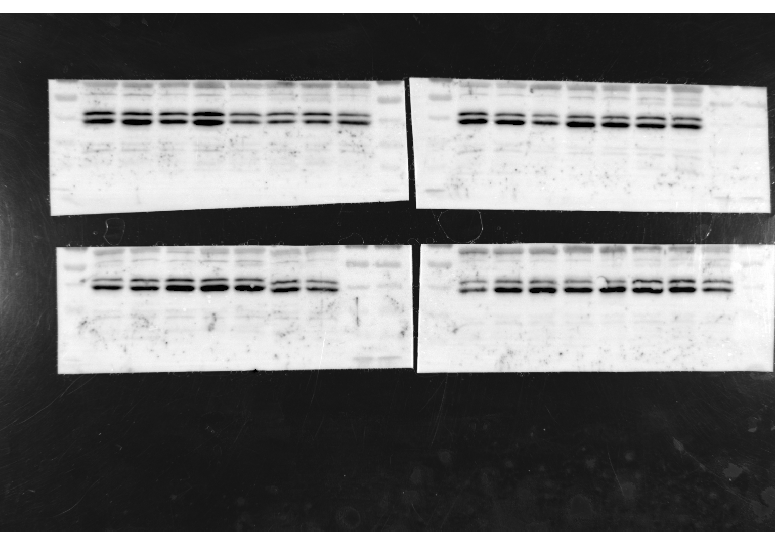


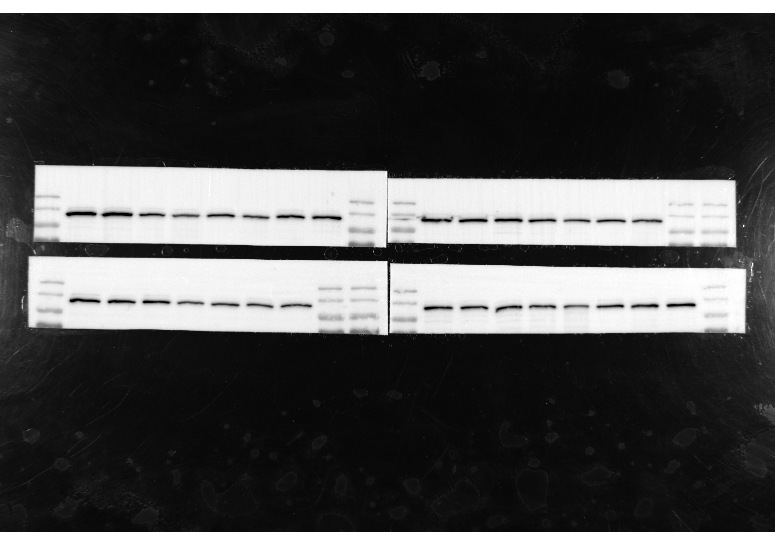


Figure S3: Plain blot membrane photographs of the proteins of Atrogin (Top), MURF (middle) and loading control Vinculin (bottom). As in the pictures before, Ctrl. is in the upper left corner, APAP 6 (removed from the study) in the upper right, APAP 12 in the lower left corner and APAP 24 in the lower right corner.


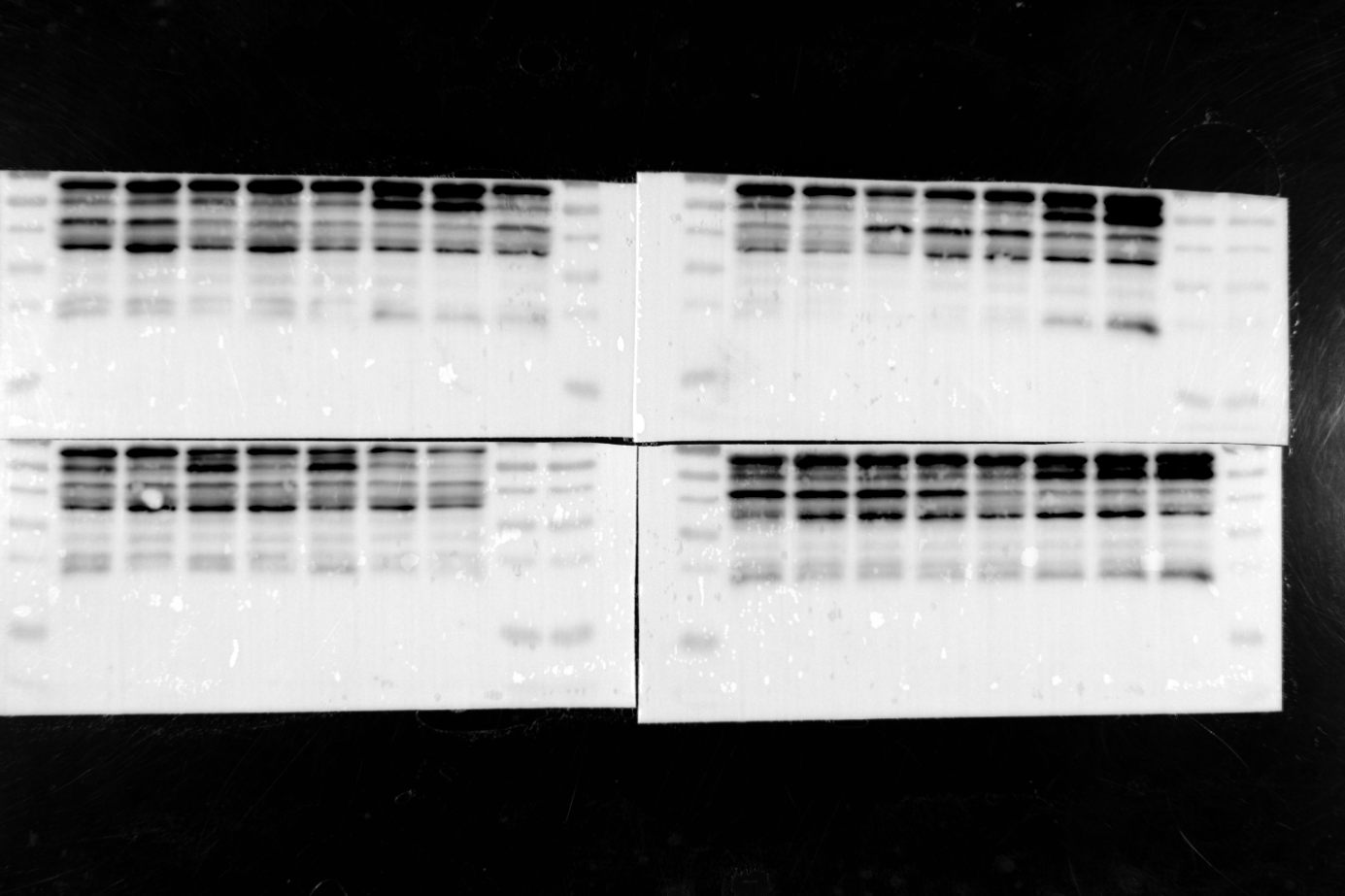


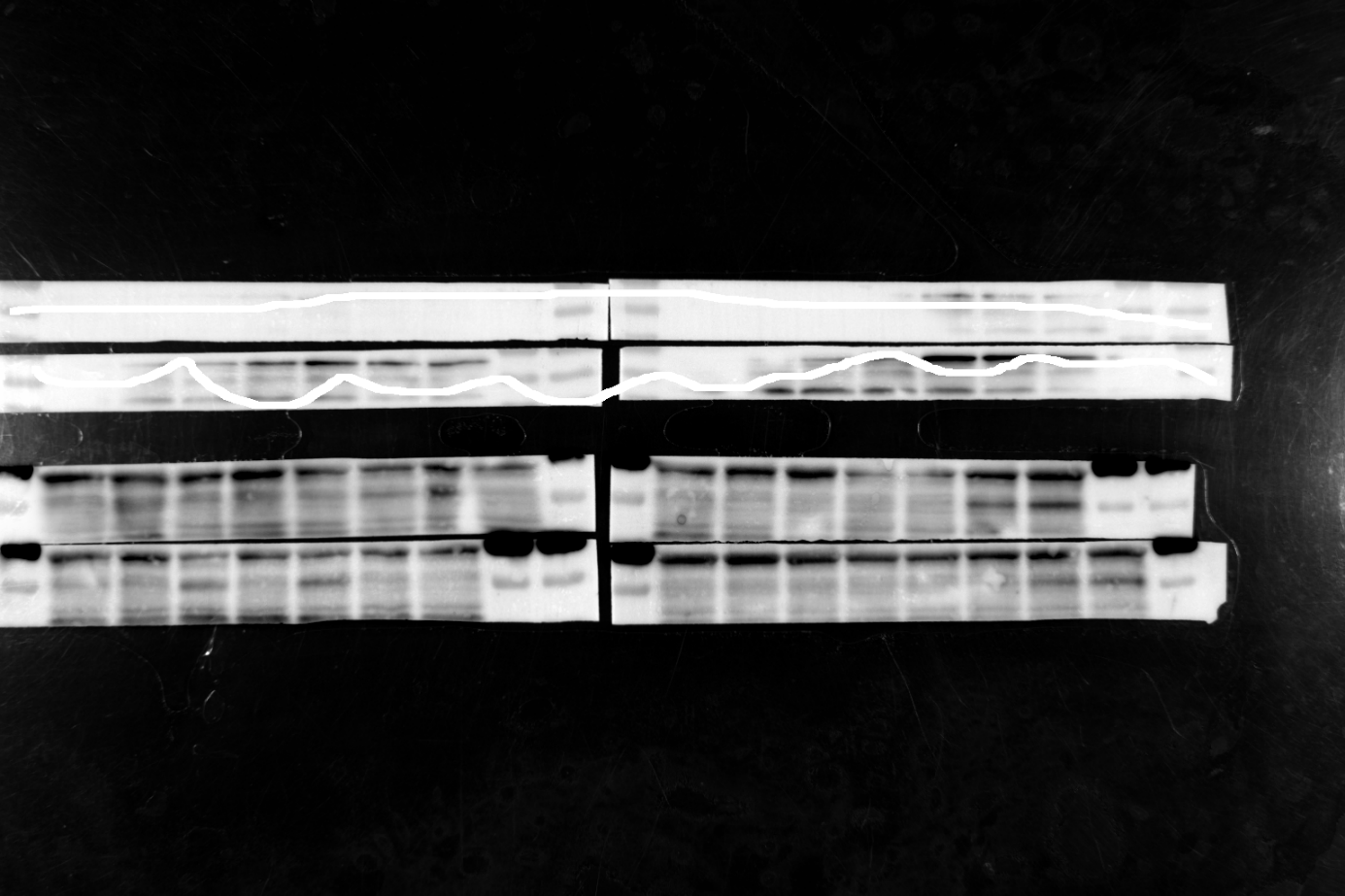


Figure S4: Plain blot membranes from p50/ p65 (top) and phosphorylated p50/p65 (bottom). The blots in the very pictures appear in the following order: Ctrl. is in the upper left corner, APAP 6 (removed from the study) in the upper right, APAP 12 in the lower left corner and APAP 24 in the lower right corner.


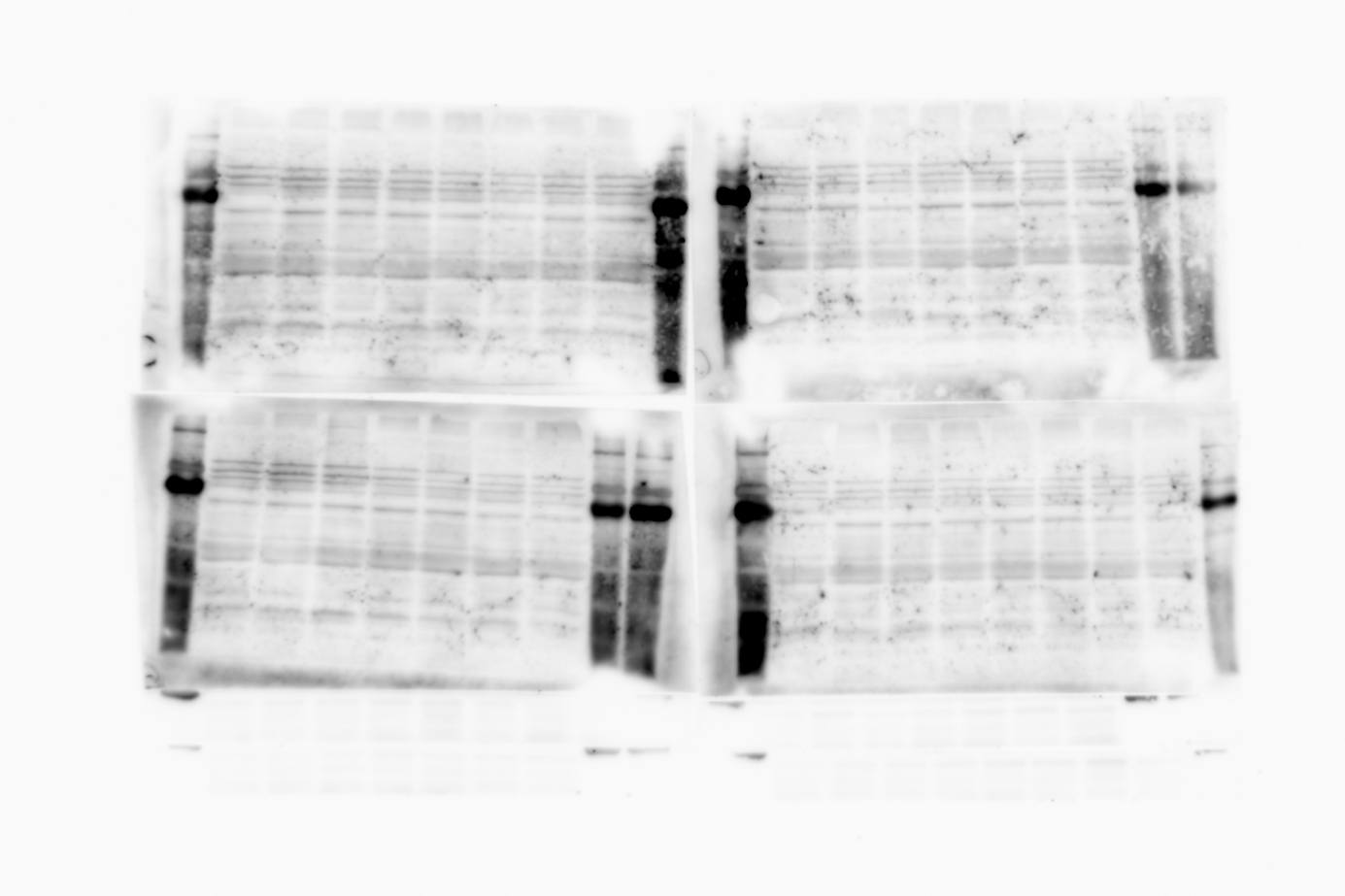


Figure S5: Membranes of 4 hydroxy-non-enal; Ctrl. is in the upper left corner, APAP 6 (removed from the study) in the upper right, APAP 12 in the lower left corner and APAP 24 in the lower right corner.

Figure S6: Blots displaying the measures for LC3B as plain photograph. The blots differ from the above mentioned order due to the fact that they were run in a different laboratory (Lab of Pneumology, Leuven).
